# Integrated One Health surveillance of human, canine and sand fly *Leishmania infantum* transmission in Sicily, southern Italy

**DOI:** 10.1186/s13071-026-07520-z

**Published:** 2026-07-11

**Authors:** Federica Bruno, Eugenia Oliveri, Francesco La Russa, Maria Liliana Di Pasquale, Roberto Hamel, Maria Clizia Di Patti, Maria Paola Maurelli, Laura Rinaldi, Gaetano Oliva, Giuseppe Pistone, Maria Rita Bongiorno, Carmelo Bongiorno, Fabrizio Vitale, Germano Castelli

**Affiliations:** 1 WOAH Leishmania Reference Laboratory, Istituto Zooprofilattico Sperimentale della Sicilia, 90129 Palermo, Italy; 2 Department of Veterinary Medicine and Animal Production, University of Naples Federico II, 80137 Naples, Italy; 3 UOC di Dermatologia e MTS, Azienda Ospedaliera Universitaria Policlinico Paolo Giaccone, 90127 Palermo, Italy

**Keywords:** Canine reservoir, *Leishmania infantum*, Leishmaniasis, Mediterranean region, One health, Sand flies, Surveillance

## Abstract

**Background:**

Leishmaniasis is an endemic vector-borne disease in Sicily, sustained by *Leishmania infantum*, with dogs acting as the primary reservoir and phlebotomine sand flies as the main vectors. Climatic changes may influence transmission dynamics, but integrated epidemiological data remain limited in several high-risk areas of the island.

**Methods:**

A One Health surveillance study was conducted between 2022 and 2024 in three Sicilian provinces (Palermo, Agrigento, and Caltanissetta). Human cases were identified through active hospital-based surveillance, and socio-demographic risk factors were assessed using structured questionnaires. Canine infection was evaluated through immunofluorescence antibody test-based serosurveys in owned and shelter dogs. Entomological monitoring was performed using CDC light traps in peri-domestic environments. Climatic variables (temperature and precipitation) were analyzed in parallel with vector captures.

**Results:**

A total of 67 confirmed human leishmaniasis cases were detected (50 cutaneous and 17 visceral), with a higher proportion of cases among outdoor workers and smokers. Canine seroprevalence ranged from 12 to 34% and spatially overlapped with human case-detection patterns. Entomological collections yielded 75,376 sand flies, with Caltanissetta accounting for 80.6% of specimens. *Phlebotomus perniciosus* was the predominant species, while *Ph. papatasi* was detected in Agrigento. Higher sand fly capture counts were observed during warm months (20–28 °C) following periods of moderate rainfall. Increasing temperature and precipitation trends coincided with the highest levels of relative sand fly activity observed in 2024.

**Conclusions:**

These findings confirm ongoing *L. infantum* transmission in Sicily and demonstrate the ecological alignment between human cases, canine reservoirs, vector captures, and climatic conditions. Climatic trends may contribute to an extension of the seasonal transmission window. Integrated veterinary and entomological surveillance can provide early signals of transmission intensification and support targeted interventions in high-risk endemic areas.

**Graphical Abstract:**

**Figure:**
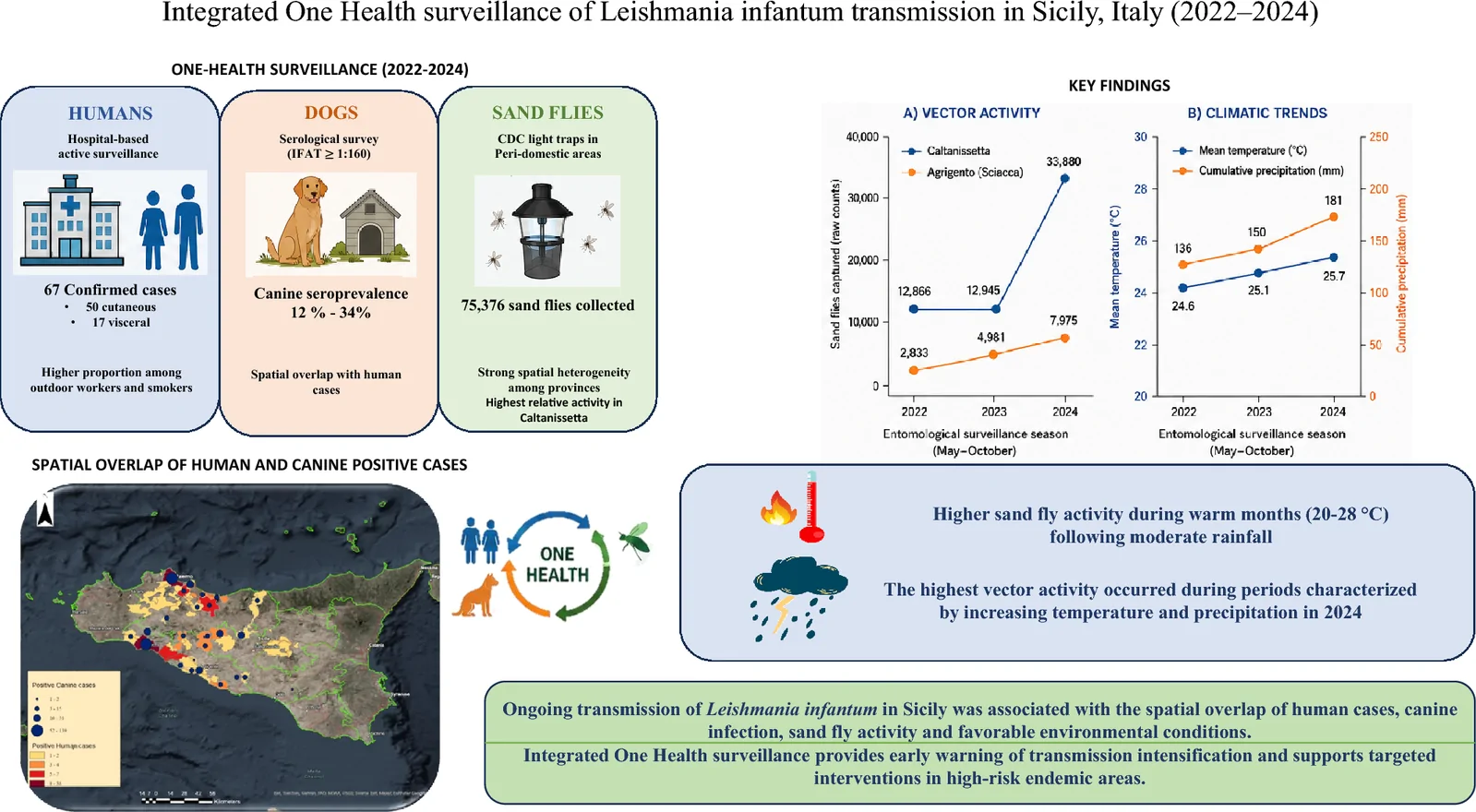
Integrated One Health surveillance of human, canine and sand fly Leishmania infantum transmission in Sicily, southern Italy

**Supplementary Information:**

The online version contains supplementary material available at https://doi.org/10.1186/s13071-026-07520-z.

## Background

Leishmaniasis is a vector-borne zoonotic disease of major public health and veterinary concern, caused by protozoa of the genus *Leishmania* and transmitted through the bite of infected female phlebotomine sand flies. In the Mediterranean basin, *Leishmania infantum* is the predominant species, responsible for both visceral (VL) and cutaneous (CL) forms in humans and for canine leishmaniasis (CanL), a chronic systemic disease in dogs. Dogs (*Canis familiaris*) act as the primary domestic reservoir and represent the main source of infection for humans, sustaining the parasite’s circulation in peri-domestic and rural environments [1–4]. In addition to dogs, cats have increasingly been recognized as potential secondary reservoirs and sentinels of *Leishmania infantum* infection in Mediterranean endemic areas. Recent studies conducted in Italy and other Mediterranean countries have documented feline exposure and infection, supporting the potential epidemiological relevance of cats within integrated One Health surveillance frameworks [5–7]. Although CanL is widespread across southern Europe, its epidemiology varies according to environmental and climatic conditions that influence sand fly vector distribution [8–11].

Sicily is a highly endemic region due to climatic conditions that facilitate the spread of both vectors and parasites. Historically, cases of canine and human leishmaniasis have been reported in central and southern Italy, regions typically characterized by mild winters and hot summers. However, in recent decades, numerous cases of both animal and human leishmaniasis have been documented in northern Italy, traditionally classified as a non-endemic area for leishmaniasis [5, 7, 15]. Similar trends have been observed in other Mediterranean countries, such as Greece [8], suggesting a geographic and ecological expansion of transmission. Although the human disease is subject to mandatory reporting, the available data are based on passive case detection, which represents only the tip of the iceberg, leading to a significant underestimation of the incidence of CL and VL. This underreporting has also been documented in other Mediterranean regions, such as the Balearic Islands, where *L. infantum*-associated cutaneous leishmaniasis remains substantially underestimated [12]. This is particularly relevant in endemic regions such as Sicily, where environmental and climatic conditions favor increased vector activity and year-round transmission cycles [9].

A retrospective analysis conducted in Sicily from 2013 to 2021 by Bruno et al. [13] found a high prevalence of CanL and elevated incidence rates of VL and CL in the human population. That study highlighted the limitations of passive surveillance and the need for an integrated One Health approach to address human, animal, and vector data simultaneously. This observation aligns with previous veterinary surveillance studies conducted in Pantelleria and other Sicilian contexts [4] reinforcing the importance of canine reservoirs in disease persistence. Similar integrated One Health approaches combining human, animal, entomological, and environmental data have also been applied in newly endemic areas of northern Italy, highlighting the usefulness of multidisciplinary surveillance frameworks for modeling transmission risk [14]. Moreover, the study of Bruno et al. [13] highlighted several gaps and challenges in the current surveillance, prevention, and control of leishmaniasis in Sicily, as well as the need to better characterize risk factors associated with both exposure and infection at local scales. Among them: uneven diagnostic coverage, fluctuations in case detection over time, limited integration of human, animal, and vector data under a One Health framework.

Recent climatic fluctuations in the Mediterranean region have been associated with changes in the distribution and abundance of sand fly vectors, particularly *Phlebotomus perniciosus* and *Phlebotomus papatasi*, potentially enhancing transmission risk in endemic areas [9, 15].

Beyond these epidemiological aspects, growing evidence indicates that climate variability plays a crucial role in shaping the transmission dynamics of *Leishmania* spp. in the Mediterranean basin. Temperature, humidity, and precipitation patterns directly affect sand fly population density, longevity, and biting activity, potentially expanding the transmission season and favoring the northward spread of the disease [9, 15, 16]. Sicily, with its heterogeneous topography and microclimatic gradients, represents a suitable case study for studying the impact of environmental and climatic drivers on leishmaniasis transmission. Integrating climatic monitoring with entomological and epidemiological data under a One Health framework is therefore essential to anticipate changes in vector ecology and to develop geographically targeted control strategies.

In light of these findings, there is still a pressing need for updated, prospective or semi‐prospective epidemiological analyses focusing on human infection risk, correlating with canine reservoir data in geographically explicit contexts, and assessing socio‐demographic, behavioral, and environmental risk factors. Our study aims to provide recent data (2022–2024) on human and canine leishmaniasis in selected Sicilian provinces (Palermo, Agrigento and Caltanissetta), analyzing risk factors (age, occupation, smoking, residence), mapping local seroprevalence patterns, and examining the correlation between canine seroprevalence and human case-detection rate at provincial and municipal levels, as well as analyzing entomological and environmental data under a One Health perspective [13, 17]. These provinces were selected because, in recent years, they have shown a marked increase in the number of reported human leishmaniasis cases, suggesting an active and possibly expanding transmission cycle. Additionally, rodents and other wild mammals have been recognized as potential secondary reservoirs in Mediterranean ecosystems, contributing to the maintenance of *L. infantum* transmission cycles [18]. This emphasizes the need for surveillance frameworks capable of integrating data from both domestic and sylvatic hosts. The aim of this study was to integrate human clinical surveillance, canine serological screening, entomological monitoring, and climatic data to describe the epidemiological patterns of *Leishmania infantum* transmission in three endemic Sicilian provinces within a One Health framework. Specifically, the study aimed to: (i) characterize human case-detection patterns, (ii) describe canine seropositivity, (iii) assess sand fly captures and species composition, and (iv) explore descriptive ecological associations between vector activity and climatic variables.

## Methods

### Study design

A descriptive observational study integrating four data streams was conducted: (i) human clinical-diagnostic surveillance (patients tested in hospital settings), (ii) targeted canine serosurveys, (iii) peri-domestic CDC light-trap sampling of sand flies, and (iv) provincial meteorological data (temperature, precipitation). Sampling windows for humans, dogs, vectors and climate are reported to document any lack of perfect synchronicity among datasets.

### Study area

Sicily, the largest island in the Mediterranean basin, was selected as the study area, with focus on the provinces of Palermo, Agrigento, and Caltanissetta. These territories present ecological and climatic conditions highly suitable for the circulation of *L. infantum* and its phlebotomine vectors. The region has a typical Mediterranean climate, characterized by hot, dry summers and mild, wet winters, with summer temperatures frequently exceeding 35–40 °C and most rainfall concentrated between autumn and early spring. Such seasonal patterns strongly influence vector activity and pathogen circulation. Sicily exhibits pronounced coastal–inland gradients and microclimatic heterogeneity that create ecological niches conducive to sand flies. Rather than emphasizing potentially imprecise altitude ranges, we focus on the main eco-epidemiological drivers—temperature, humidity, vegetation cover, and peri-urban rural interfaces—which jointly modulate *Leishmania* transmission dynamics across the study area. Land use is heterogeneous, transitioning from densely populated coastal urban centers (e.g., Palermo) to agricultural and rural inland areas (e.g., Agrigento and Caltanissetta), where environmental conditions and animal reservoirs favor stable sand fly populations. These climatic and landscape characteristics, coupled with increasing temperatures and irregular precipitation trends observed in recent years, support persistent vector circulation and may contribute to seasonal extension and spatial expansion of transmission hotspots.

### Samples

#### Human survey

Human screening was conducted at the University Hospital “P. Giaccone” in Palermo and collaborating infectious disease and dermatology units in the provinces of Agrigento and Caltanissetta. Both symptomatic and asymptomatic individuals were enrolled after providing written informed consent. Patients were enrolled because they were referred to the participating Infectious Diseases or Dermatology Units for clinical evaluation, diagnostic investigation, or follow-up in the context of suspected leishmaniasis or other infectious diseases surveillance activities. Each participant completed a structured questionnaire collecting socio-demographic data, clinical history, and behavioral and lifestyle variables potentially associated with exposure risk for *Leishmania* infection (including occupation, smoking habits, ownership or contact with animals, outdoor activities, and municipality of residence). Biological specimens were obtained according to clinical presentation: blood and/or bone marrow aspirates for suspected VL, skin biopsies or lesion samples for suspected CL. All samples were processed under accredited diagnostic protocols. PCR and/or indirect immunofluorescence antibody test (IFAT) were performed for *L. infantum* detection following standard operating procedures. Serological testing was used as a complementary diagnostic tool, while case confirmation was primarily based on molecular detection. A substantial proportion of enrolled subjects derived from Infectious Diseases Unit surveillance activities involving HIV-positive individuals. Overall, 433 enrolled patients were HIV-positive and followed within the Infectious Diseases Unit surveillance program.

Specimens were obtained from specialist hospital-based clinical settings; therefore, the human dataset reflects individuals who were clinically tested rather than the general population. As a result, the metric reported represents a clinical detection rate, not a true population incidence. Any comparative analyses using resident population denominators are exploratory and should be interpreted with caution.

#### Canine survey

Following identification of a human leishmaniasis case, targeted active surveillance was conducted in the surrounding canine population. Sampling included privately owned dogs and shelter dogs from the same municipality, ensuring representation of domestic and peri-urban canine populations. In municipalities reporting human cases, at least 20 dogs were sampled whenever possible, resulting in the examination of 3,046 dogs over the study period. For each dog, anamnestic and clinical data were collected (sex, age, lifestyle, municipality), and serum and blood and/or lymph node aspirates were obtained. This response-based, non-probabilistic sampling design was intended to identify local reservoir activity around human cases. As such, resulting seroprevalence values should be interpreted as contextual surveillance estimates, not as population-wide prevalence rates, and primarily serve to support integrated human–animal–vector risk assessment.

### Diagnosis

*Leishmania* infection was investigated using both serological and molecular approaches. An indirect immunofluorescence antibody test (IFAT) was applied to sera from human and canine samples, following the guidelines of the WOAH manual. Serum dilutions were performed in duplicate, and titers ≥ 1:160 were considered positive. Positive and negative controls were included in each run. Molecular diagnosis was carried out by Real-Time PCR targeting *L. infantum* DNA. For suspected VL, DNA was extracted from blood and/or bone marrow; for suspected CL, tissue or skin biopsy samples were analyzed; and for dogs, lymph node aspirates were tested. PureLink™ Genomic DNA Mini Kit (Thermo Fisher Scientific K182002, Waltham, MA, USA) was used according to the manufacturer’s instructions. Real-time PCR was performed using a LightCycler® 96 (Roche Life Science) following standardized protocols established by C.Re.Na.L [19, 20].

### Vector survey

Entomological surveillance was conducted in parallel with human and canine investigations to monitor *L. infantum* vector presence and relative activity. Sand flies were sampled from May to October during 2022–2024 using CDC miniature light traps placed in peri-domestic environments of municipalities under study. Trapping sites were selected based on prior reports of human or canine leishmaniasis and ecological suitability (vegetation, animal shelters, organic debris, shaded areas). Traps were deployed approximately 1.5 m above ground level and operated overnight from dusk to dawn. Sampling effort was recorded and expressed as trap-nights, allowing descriptive comparison of relative sand fly activity across sites and periods. Among the total 383 trap-nights performed during the study period, 212 conducted during the main entomological season (May–October) were included in the standardized comparative analysis presented in this study. Accordingly, the higher values reported for Palermo (214), Agrigento (38), and Caltanissetta (131) refer to total trapping effort across the full study period, whereas the standardized comparative analyses and Supplementary Table S3 refer exclusively to trap-nights performed during the May–October surveillance season.

Collected sand flies were preserved in 70% ethanol and transported to the laboratory. Male specimens were identified morphologically to species level using established taxonomic keys, while female specimens were sorted by species and collection site and examined for feeding status. Female pools were preserved for molecular analysis where applicable, although PCR-based xenomonitoring results are not included in the present report. Because sampling effort was recorded, it was not fully standardized across all sites and years due to logistical and environmental constraints, comparisons of sand fly captures should be interpreted as indicative of relative activity rather than directly comparable densities. Sampling effort was higher in Palermo compared to the other provinces, yet sand fly captures remained markedly lower.

### Climatic and environmental data collection

Climatic data were obtained from the *Servizio Informatico Agrometeorologico Siciliano* (SIAS), the regional agrometeorological monitoring network. Monthly temperature (°C) and precipitation (mm) from meteorological stations located near the study areas in Palermo, Agrigento, and Caltanissetta were retrieved for 2022–2024.

For each province, climate data were aligned with the entomological sampling period (May–October) to evaluate seasonal trends and their relationship with sand fly abundance. To account for potential delayed meteorological effects on vector dynamics, climatic variables were examined not only contemporaneously but also considering short-term lagged patterns at the descriptive level. Trends were compared with seasonal and interannual variation in sand fly captures to support ecological interpretation of sand fly population dynamics in the study area.

### Ethics statement

Human data and biological samples were collected within an approved observational study conducted in accordance with the Declaration of Helsinki and relevant national regulations.

The study protocol *“Leishmaniasis in Agrigento, Caltanissetta and Palermo provinces: human outbreaks and animal reservoirs”* (Prot. GR-2019–12369134) was reviewed and approved by the Ethics Committee Palermo 1 (Azienda Ospedaliera Universitaria Policlinico “P. Giaccone”, Palermo; approval no. 04/2021, session of 28 April 2021). All participants provided written informed consent prior to enrollment, and data were anonymized before analysis.

Canine sampling was performed as part of official veterinary surveillance and diagnostic activities, in compliance with Italian and European legislation on animal welfare. Sampling was carried out within routine clinical practice after obtaining informed consent from dog owners. No experimental procedures were conducted on animals specifically for this study, and no sedation or procedures other than those routinely applied in clinical practice were performed.

### Statistical analysis

Data analysis was performed using GraphPad Prism (version 9.5.1, GraphPad Software, San Diego, CA, USA) and SPSS® software (version 30.0, IBM Corporation, Armonk, USA). Descriptive statistics were used to summarize demographic, clinical, and epidemiological variables. Human case-detection rate was expressed as cases per 100,000 inhabitants, while canine seroprevalence was calculated as the proportion of seropositive animals among the total tested. Associations between categorical variables (e.g., age, smoking habits, occupation, residence) and *Leishmania* infection were evaluated using the Chi-square test. Correlations between canine seroprevalence and human case-detection rate were assessed using Spearman’s rank correlation coefficient (*ρ*). A significance threshold of *P* < 0.05 was adopted for all statistical tests. Given the observational and ecological design of the study, statistical analyses were primarily exploratory and descriptive in nature.

## Results

### Human cases

From 2022 to 2024, a total of 559 human samples were analyzed in the Sicilian provinces of Palermo, Agrigento, and Caltanissetta. Of these, 461 samples originated from infectious disease departments and 98 from dermatology units. Overall, 67 out of 559 samples tested positive for *Leishmania infantum*, corresponding to an observed positivity rate of 11.98%. Among the confirmed cases, 50 (74.6%) were CL and 17 (25.4%) VL. Among the 433 HIV-positive individuals included in the surveillance cohort, 15 were diagnosed with leishmaniasis during the study period. Detailed demographic and epidemiological characteristics of the screened human population are summarized in Supplementary Table S1.

Human cases were unevenly distributed across the three provinces. Palermo accounted for the majority of cases, with 46/67 (68.7%) patients, followed by Agrigento with 20/67 (29.9%), and Caltanissetta with 1/67 (1.49%). Most CL cases presented with typical ulcerated lesions characterized by well-defined margins, whereas VL cases were confirmed through bone marrow aspirates and PCR.

Temporal distribution showed a seasonal pattern, with most diagnoses occurring between late spring and early autumn. Infections were more frequent in the 51–70-year age group ((50.7%; *χ*^2^ = 16.89, *P* = 0.002) and males were more commonly affected than females. Occupational exposure appeared to be an important risk factor: outdoor workers such as farmers, construction workers, and forestry personnel represented a large proportion of the cases (*P* < 0.001, *χ*^2^ = 31.536). Behavioral risk factors were also investigated, and smoking emerged as a significant predictor of PCR-positivity (*χ*^2^ = 4.26, *P* = 0.039), suggesting that lifestyle factors may increase susceptibility to infection.

### Canine cases

Parallel surveillance of the canine population revealed widespread infection. Out of 3,046 dogs tested, seroprevalence rates ranged between 12 and 34% depending on the municipality of origin. The highest prevalences were detected in Agrigento and Palermo provinces, which were also the areas with the largest clusters of human cases. Dogs from both rural and peri-urban settings were affected, including privately owned dogs and sheltered dogs, confirming the broad distribution of infection among different canine groups. No significant differences were observed between males and females, although younger animals (< 2 years) showed lower positivity rates compared to adults, consistent with cumulative exposure over time. The overlap between canine hotspots and areas of human case-detection rate strongly supports the role of dogs as the primary reservoir sustaining transmission. Detailed canine IFAT results by municipality and province are summarized in Supplementary Table S2.

### Human case-detection rate and correlation with canine seroprevalence in the study area

**Fig. 1:**
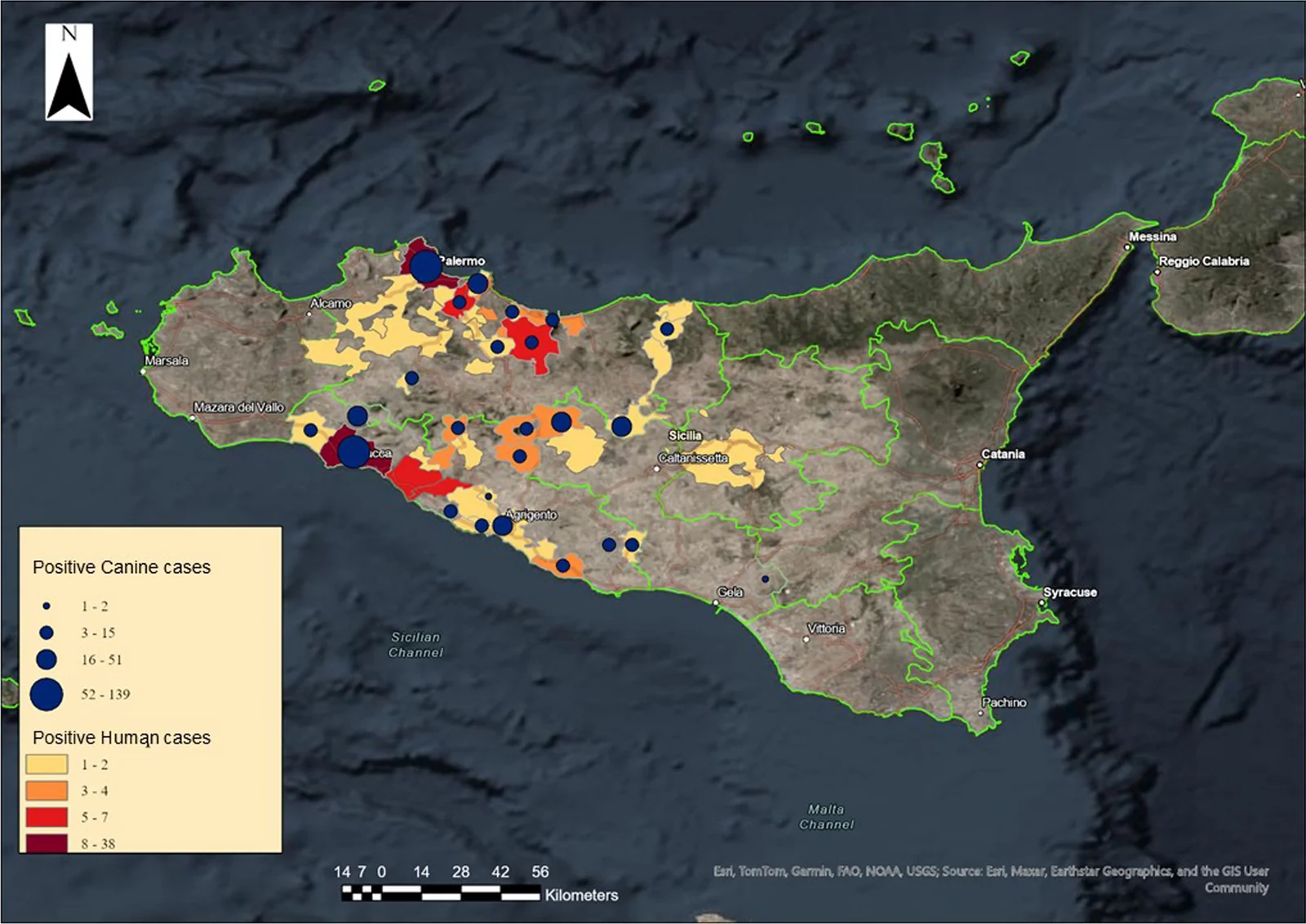
Spatial distribution of confirmed human leishmaniasis cases and seropositive dogs in the study area (Palermo, Agrigento, and Caltanissetta provinces), 2022–2024. Human cases are represented as choropleth classes at the municipal level, while canine cases are shown as proportional symbols. The map highlights areas of spatial overlap as well as heterogeneity between human and canine infection patterns and is intended for descriptive and exploratory purposes. The map was generated using QGIS software (QGIS Development Team)

**Fig. 2:**
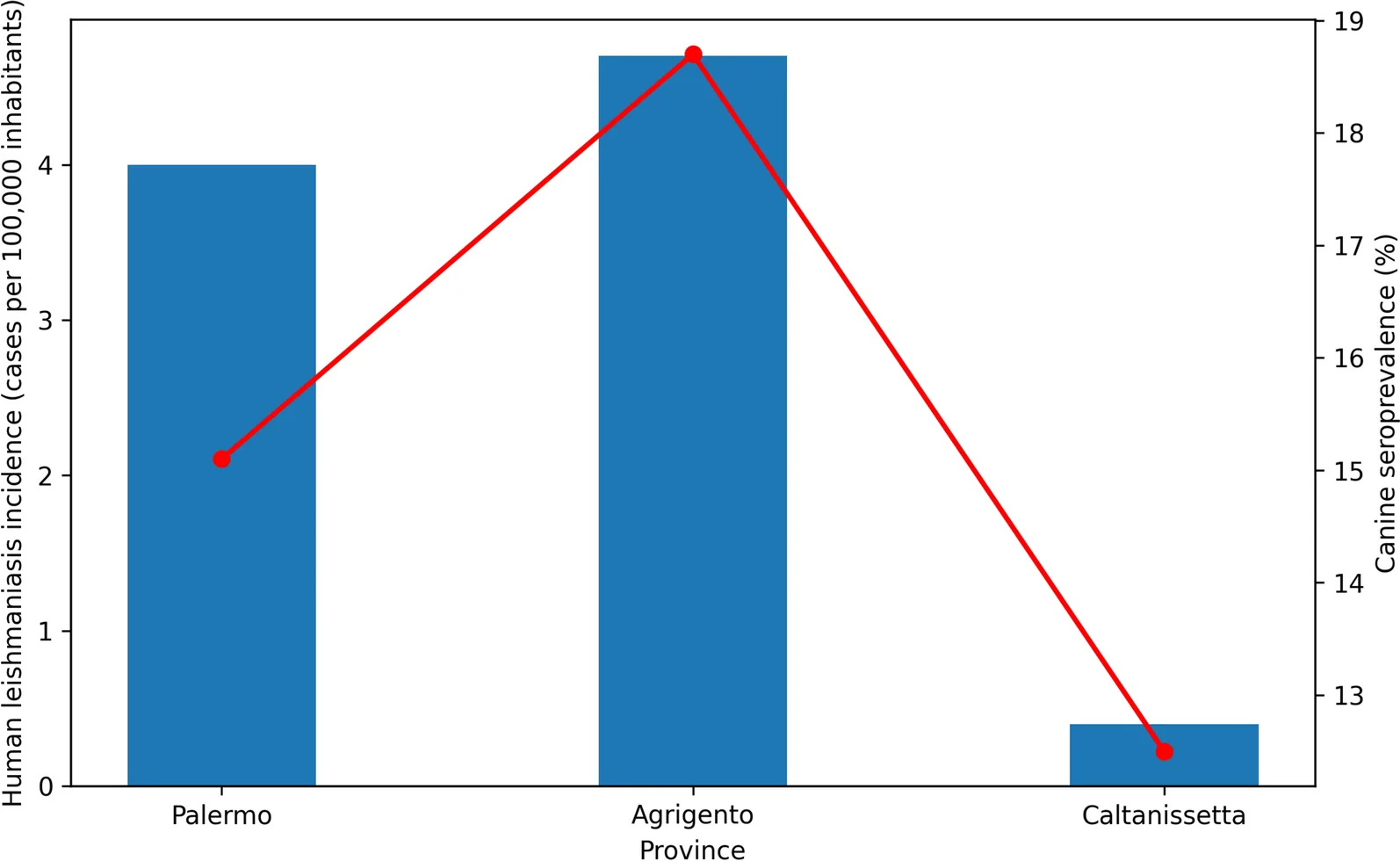
Comparison of human case-detection rate and canine seroprevalence by province in Sicily (2022–2024). Bars represent human case-detection rate expressed as cases per 100,000 inhabitants, while the line indicates canine seroprevalence (%). The figure highlights provincial heterogeneity and descriptive concordance between human and canine infection patterns within a One Health framework

The observed prevalence of human leishmaniasis among screened patients was 12% (67/559), with marked variation across provinces (Fig. 1). To estimate true epidemiological impact, PCR-confirmed cases were also expressed as incidence per 100,000 inhabitants using ISTAT 2022 population data. Agrigento reported 4.7 cases/100,000 inhabitants (20 cases in ~ 422,000 residents), Palermo 3.9 cases/100,000 (46 cases in ~ 1,196,000 residents), and Caltanissetta 0.4 cases/100,000 (1 case in ~ 256,000 residents), reflecting higher transmission intensity in Agrigento and Palermo compared to Caltanissetta. A comparison between human case-detection rate and canine seroprevalence at the provincial level is shown in Fig. 2.

When correlating human case-detection rate with canine seroprevalence, a strong positive association was observed at the provincial level (Spearman’s *ρ* = 1.00). However, given the very limited number of provinces included (*n* = 3), robust statistical inference was not possible and this finding should be interpreted as descriptive and ecological. At the municipal level, no significant correlation was observed (*ρ* = −0.03, *P* = 0.91), likely reflecting small sample sizes, local ecological heterogeneity, and variability in human exposure patterns. Despite these limitations, the geographical overlap between canine seroprevalence hotspots and human case clusters reinforces the epidemiological importance of dogs as the primary reservoir sustaining *L. infantum* transmission and highlights the utility of canine surveillance as a complementary sentinel tool for human risk assessment in endemic settings.

### Entomological results

A total of 75,376 sand flies were collected between 2022 and 2024. Detailed entomological captures, trap-night distribution, and associated decadal climatic variables are summarized in Supplementary Table S3.

The number of captured specimens increased progressively over the study period: 16,309 in 2022, 17,212 in 2023, and 42,355 in 2024, reflecting a substantial rise in sand fly captures during the last year of surveillance.

Marked geographical heterogeneity was observed. Caltanissetta province accounted for the great majority of specimens (60,766; 80.6%), followed by Agrigento (15,044; 19.9%), while only 66 specimens (0.09%) were collected in Palermo during the entire period.

Regarding sex distribution, both males and females were trapped, with an overall predominance of males in 2024. A small number of engorged females was also recorded each year, indicating the presence of potentially infectious sand flies in the surveyed areas.

Morphological identification confirmed that *Phlebotomus perniciosus* was the predominant species, followed by *Ph. perfiliewi* and *Sergentomyia minuta*. In Agrigento, the presence of *Ph. papatasi* was also documented.

The exceptionally high abundance of sand fly capture counts in Caltanissetta, and to a lesser extent in Agrigento, mirrors the geographical distribution of human and canine cases, showing a spatial overlap with areas reporting higher numbers of human and canine cases.

### Climatic correlations and vector dynamics

To better interpret vector distribution, climatic data provided by the Sicilian Agrometeorological Information Service (SIAS) were analyzed in parallel with entomological findings. This comparison allowed the correlation of sand fly captures with local environmental conditions across the three provinces. Temperature and precipitation data revealed microclimatic differences among the study areas, which were associated with variations in sand fly captures and seasonal activity.

In particular, the Agrigento area exhibited mild temperatures for most of the year and moderate yet constant rainfall, conditions consistent with increased sand fly captures. Consistently, this province recorded a high number of sand flies captured (> 3,700 specimens), in line with the higher numbers of human cases and canine seropositive dogs observed. Conversely, the Palermo area, characterized by an urban and coastal microclimate with stronger temperature fluctuations and lower ambient humidity, yielded a much smaller number of captures (66 specimens) despite broader sampling coverage.

Seasonal trends in vector captures closely overlapped with climatic patterns. Higher sand fly captures occurred during months with average temperatures between 20 and 28 °C and moderate humidity levels, typically from late spring to early autumn. During winter, the sharp temperature drop and limited availability of moist microhabitats led to a marked reduction in vector presence. Overall, SIAS data highlighted spatial and temporal concordance between climatic variability and sand fly captures across the study areas, supporting the usefulness of integrated entomological and meteorological surveillance within a One Health framework. The integration of entomological surveillance and meteorological monitoring thus represents an essential approach to understanding epidemiological dynamics and guiding targeted prevention strategies at the local level.

During the main entomological season, defined as May–October, 74,783 sand flies were collected over 212 documented trap-nights. When captures were standardized by sampling effort, marked spatial and temporal heterogeneity emerged across provinces. Caltanissetta showed the highest relative sand fly activity over the three-year period, with 59,674 sand flies collected over 64 trap-nights, corresponding to 932.4 captures per trap-night. Agrigento also showed high relative activity, with 15,043 sand flies collected over 33 trap-nights, corresponding to 455.8 captures per trap-night. In contrast, Palermo showed persistently low relative activity, with only 66 sand flies collected over 115 trap-nights, corresponding to 0.6 captures per trap-night.

**Fig. 3:**
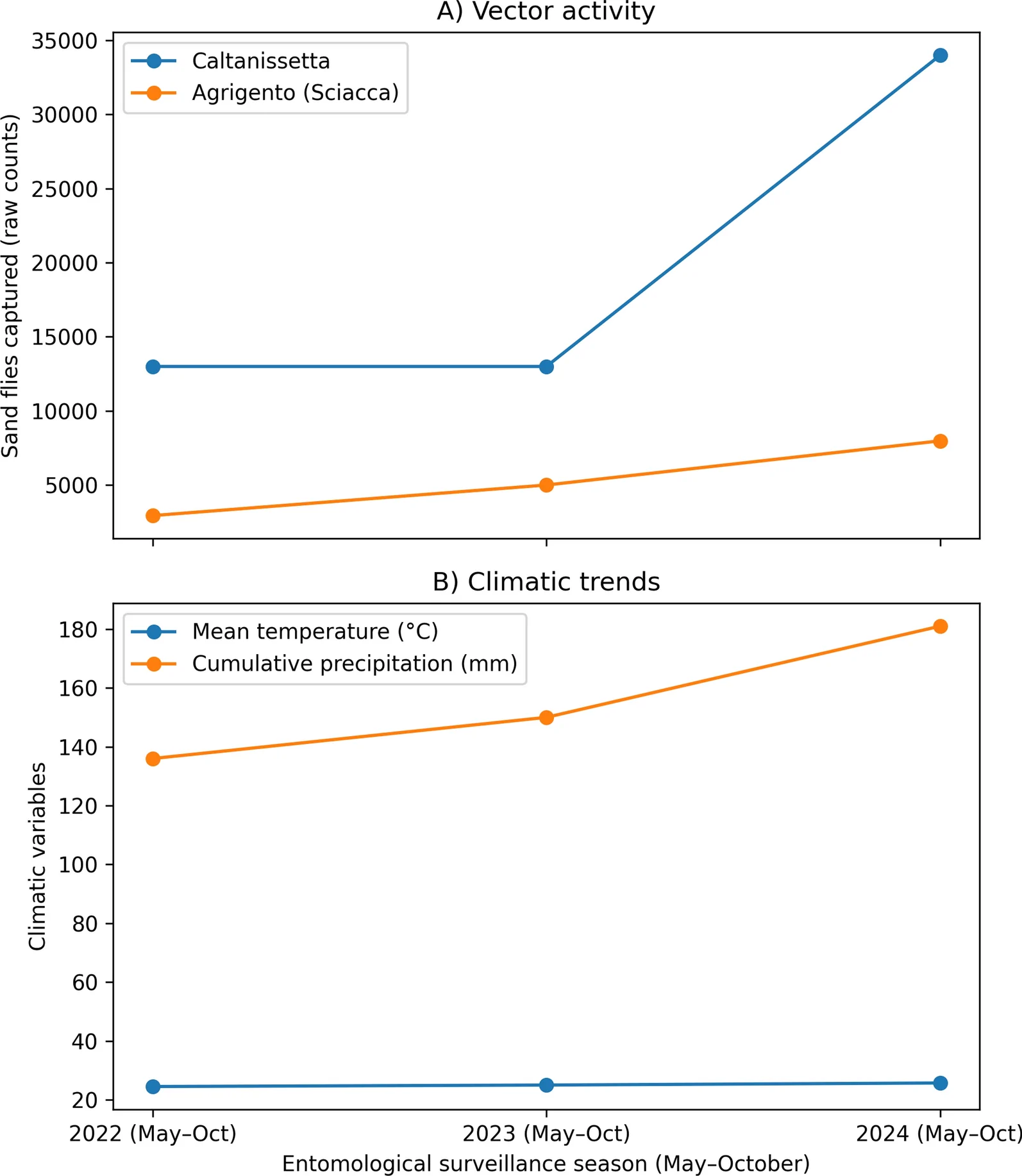
Temporal trends in vector activity and climatic conditions during entomological surveillance seasons (May–October, 2022–2024). **A** Number of sand flies captured, expressed as raw counts, in Caltanissetta and Agrigento (Sciacca). **B** Mean temperature and cumulative precipitation recorded during the same surveillance periods. Panels illustrate descriptive temporal concordance between climatic conditions and sand fly activity

The highest annual capture rate was observed in Caltanissetta in 2024, where 34,313 sand flies were collected over 27 trap-nights, corresponding to 1,270.9 captures per trap-night. This increase was temporally concordant with higher cumulative precipitation in the matched climatic decades, which reached 281.0 mm in 2024 compared with 150.4 mm in 2022 and 135.6 mm in 2023, while temperatures remained within seasonal ranges previously associated with sand fly activity. However, the persistently low capture rates observed in Palermo despite extensive trapping effort suggest that climatic variables alone may not fully explain local variation in sand fly captures, indicating that additional ecological and environmental factors could also influence vector presence (Fig. 3).

### Study limitation

This study has some limitations. The inclusion of only three provinces reduced statistical power for some correlation analyses, particularly those involving climatic variables. Both human and canine sampling followed an observational, non-probabilistic design, potentially introducing selection bias. Although trap-night standardization was applied, entomological data may still be subject to sampling-effort bias, and xenomonitoring parameters such as minimum infection rates (MIR) and blood-meal source analysis were not performed, limiting inference on vector infection and host-feeding patterns. In humans, data were derived from clinically tested individuals rather than from population-wide notification systems, meaning that results reflect a clinical detection rate rather than true incidence. Because a substantial proportion of screened individuals were derived from HIV-focused Infectious Diseases Unit surveillance activities, the hospital-based cohort may not fully reflect the epidemiological structure of the general population. Behavioral and socio-economic confounders (e.g., repellent use, housing characteristics) were not systematically recorded, which may have influenced risk estimations. In the canine population, detailed information regarding the proportion of kennel versus privately owned dogs and the systematic use of sand fly preventive measures (e.g., insecticidal collars or topical repellents) was not uniformly available across all municipalities. These factors may have influenced local seroprevalence estimates and should therefore be considered potential sources of heterogeneity in the interpretation of canine infection patterns. Finally, the climate–vector assessment relied on a relatively short time series; thus, ecological associations should be interpreted cautiously and validated through longer longitudinal datasets and mechanistic models in future studies.

## Discussion

This study provides updated epidemiological data on human, canine, and vector leishmaniasis in three Sicilian provinces, framed within a One Health perspective. By integrating clinical surveillance, veterinary monitoring, and entomological data, our findings are consistent with ongoing *L. infantum* transmission in an endemic setting and highlight operational implications for surveillance and control, underscoring the need for strengthened, integrated public health strategies in the region. The detection of 67 confirmed human cases, predominantly cutaneous leishmaniasis, reflects the endemic nature of the disease and represents a clinical-detection rate in a hospital-tested cohort, underscoring the value of active surveillance strategies in identifying cases that may otherwise go unreported. An association between smoking and PCR positivity (*P* = 0.039) was observed, suggesting a potential behavioral component to infection risk. However, this finding should be interpreted cautiously as hypothesis-generating, given possible residual confounding (e.g., age, sex, outdoor work, dog contact) and the exploratory nature of the analysis. A speculative biological explanation for the observed association between smoking and infection may involve increased carbon dioxide (CO_2_) emissions and the release of volatile organic compounds (VOCs), both of which are recognized attractants for phlebotomine sand flies. However, this hypothesis requires further investigation and should be interpreted with caution. The use of CO_2_ as an attractant has been well documented in multiple entomological studies and is routinely exploited in trapping methods, suggesting that increased CO_2_ and VOC emissions in smokers may enhance their exposure to the vector. Conversely, although a higher proportion of cases was observed among individuals engaged in outdoor occupations, this pattern did not reach statistical significance and should therefore be interpreted as a trend that warrants further investigation in larger cohorts. These results are consistent with previous reports from southern Europe, where occupational and peri-domestic exposure play a central role in transmission. Similar One Health frameworks have been applied in other Mediterranean settings, highlighting the value of integrating human, animal, and environmental data for improved surveillance and control [13, 17, 21]. Although HIV infection has historically represented an important risk factor for visceral leishmaniasis, the widespread use of effective antiretroviral therapy has substantially changed the epidemiological profile of HIV/Leishmania coinfection in Mediterranean countries. In our hospital-based cohort, leishmaniasis positivity was not concentrated among HIV-positive individuals; however, data on CD4 + T-cell counts, HIV viral load, antiretroviral treatment status, and duration of HIV infection were not systematically available. Therefore, the role of HIV-related immunosuppression could not be formally assessed and should be interpreted cautiously.

Canine seroprevalence (12–34%) further confirms the role of dogs as the primary domestic reservoir. The spatial overlap between canine seropositive hotspots and human clinical-detection clusters —particularly in Palermo and Agrigento—reinforces the epidemiological coherence of the One Health model. Although the correlation between human and canine infection rates was descriptive at the provincial level, the pattern mirrors observations in other Mediterranean endemic areas, consistent with previous findings documenting the expanding role of dogs as a primary reservoir across Italy and other endemic regions [2, 15, 22]

Entomological findings strengthen this framework. Collections exceeded 75,000 sand flies over the three-year period, with Caltanissetta accounting for more than 80% of specimens. This pronounced spatial heterogeneity partially overlaps with the distribution of human and canine cases. However, because sampling effort was not fully standardized across all sites and years, these counts should be interpreted as indicators of relative sand fly activity rather than directly comparable densities. *Phlebotomus perniciosus* was confirmed as the dominant species, consistent with its known role as the primary vector in southern Europe, in line with previous entomological studies documenting the predominance and seasonal activity of *Ph. perniciosus* in endemic foci of Italy and France [23, 24]. Importantly, the detection of *Ph. papatasi* in Agrigento [13] may reflect shifts in community composition; implications for local *L. infantum* transmission remain uncertain. The presence of engorged females, albeit in low numbers, demonstrates active blood feeding and confirms the transmission potential of local sand fly populations. An illustrative example of this complexity is provided by the province of Caltanissetta, where more than 60,000 sand flies were collected despite recording one of the lowest human case-detection rates (0.40 cases/100,000 inhabitants). This apparent discrepancy indicates that sand fly captures alone are insufficient to explain the epidemiological impact on the human population. Several factors may account for this phenomenon. First, entomological captures were performed in ecologically favorable sites (e.g., kennels and humid rural environments) that may not accurately reflect residential exposure levels. In addition, the general population in Caltanissetta may experience lower contact rates with vectors due to reduced population density and predominantly indoor occupational activities. Moreover, underdiagnosis cannot be excluded, as mild cutaneous forms may go unrecognized or unreported, leading to an underestimation of the true disease burden. Overall, the Caltanissetta case highlights that leishmaniasis transmission arises from the interplay of multiple factors—including sand fly captures, reservoir prevalence, human exposure, and diagnostic capacity. Extending the temporal perspective to the preceding period (2015–2021), SIAS data showed that Sicilian summers were characterized by lower rainfall (100–120 mm in Caltanissetta), though temperatures had already been rising. Since 2020, a progressive increase in both summer rainfall and heat has likely predisposed the environment for the subsequent vector population boom observed between 2022 and 2024. This pattern suggests that interannual climatic variations, acting synergistically across environmental variables, may be associated with variations in sand fly activity and consequently with changes in local epidemiological risk of leishmaniasis. Our entomological results are broadly consistent with previous observations from Sicily. Bruno et al. [13] reported 467 human cases between 2013 and 2021—predominantly cutaneous (71%) rather than visceral (29%)—with Agrigento (45.4%) and Palermo (37%) identified as the most affected provinces. Their entomological analysis identified *Phlebotomus perfiliewi* as the predominant species, followed by *Ph. perniciosus* and *Sergentomyia minuta*, with the emergence of *Ph. papatasi* in Agrigento.

In our study, *Ph. perniciosus* was the dominant species, whereas *Ph. perfiliewi* was not detected in our collections, which may reflect differences in sampling sites, trapping periods, and ecological microhabitats rather than true absence. Both analyses identified Agrigento and Palermo as provinces with higher transmission potential, albeit with distinct vector dynamics. The detection of *Ph. papatasi* in Agrigento may indicate an expansion of vector species involved in transmission, warranting further investigation.

Importantly, vector dynamics closely reflected climatic trends. Warmer temperatures (20–28 °C) and moderate precipitation during spring and summer coincided with peak sand fly activity. Both temperature and precipitation displayed an upward trend in recent years, with 2024 representing the warmest and wettest year of the study period—corresponding to the highest vector captures. These findings align with previous studies demonstrating that climatic fluctuations strongly influence sand fly population dynamics and may extend the seasonal transmission window. While our results are descriptive, they are consistent with broader evidence of climate-driven modulation of vector activity; formal assessment of delayed and non-linear effects will require distributed-lag modeling on longer time series. This also highlights the seasonal nature of transmission in Sicily, with the risk period potentially lengthening under ongoing climate change.

The observed geographical and temporal overlap between high canine seroprevalence, human case-detection rate, and vector captures carries clear public health implications. Veterinary surveillance may serve as an early-warning sentinel for human risk, enabling more timely and geographically targeted control measures. The integrated One Health approach applied here represents a practical model for managing complex zoonoses, emphasizing the interconnectedness of human, animal, and environmental health.

This framework requires closer collaboration between clinicians, veterinarians, entomologists, and public health authorities, fostering coordinated surveillance networks capable of jointly collecting and analyzing epidemiological, entomological, and climatic data. Coordinated networks that jointly analyze epidemiological, entomological and climatic data can support scenario-based alerts and resource allocation. Such integration can help anticipate seasonal peaks and target control measures geographically, enhancing their effectiveness.

The limited number of provinces reduced the statistical power of correlation analyses. Variability in the sampled canine population and the lack of detailed human behavioral exposure data may have introduced selection bias and led to an underestimation of some risk factors.

Looking ahead, several methodological and operational priorities emerge. The observed increases in temperature and rainfall, paralleled by highest number of sand flies captured, suggest a lengthening of the transmission season and potential expansion of foci. Future work should incorporate probability-based canine sampling to strengthen population inference, standardized entomological effort with infection indicators (e.g., minimum infection rate, blood-meal analysis), and geospatial alignment of human exposures with trapping sites. Developing spatial–temporal predictive models, including distributed-lag and non-linear frameworks, will be essential to support early warning and targeted interventions. These advances, integrated within a strengthened One Health surveillance network, can enhance preparedness and reduce the future burden of leishmaniasis in Sicily.

## Conclusions

This study provides an integrated overview of human, canine, entomological, and climatic data related to *Leishmania infantum* transmission in Sicily within a One Health framework. The observed overlap between human case-detection patterns, canine seropositivity, and sand fly activity supports the value of integrated surveillance in endemic Mediterranean settings. Climatic variability and heterogeneous vector activity may contribute to shaping local transmission dynamics, although these ecological associations remain descriptive and require confirmation through longer longitudinal studies. Strengthening coordinated human, veterinary, and entomological surveillance may improve early detection and support targeted prevention strategies in areas at higher epidemiological risk.

## Supplementary Information

Additional file1 (DOCX 19 KB)

Additional file2 (DOCX 25 KB)

Additional file3 (DOCX 20 KB)

## Data Availability

Data supporting the main conclusions of this study are included in the manuscript.
