# Supplementary material for: Integrated One Health surveillance of human, canine and sand fly Leishmania infantum transmission in Sicily, southern Italy
Source: Parasit Vectors. 2026 Jul 11;19:431. doi: 10.1186/s13071-026-07520-z (PMC13640383; doi:10.1186/s13071-026-07520-z)
Supplement: Supplementary file 1 [file 13071_2026_7520_MOESM1_ESM.docx]

**Supplementary Table S1**. Demographic and epidemiological characteristics of screened human subjects and confirmed positive cases in Sicily (2022–2024).

| **Variable** | **Category** | **Total screened (n)** | **Positive (n)** | **PCR positivity** | **IFAT positivity** |
| --- | --- | --- | --- | --- | --- |
| **Age group** | 0–20 years | 13 | 5 | 5 | 0 |
|  | 21–50 years | 204 | 20 | 19 | 1 |
|  | 51–70 years | 287 | 35 | 34 | 1 |
|  | >70 years | 55 | 7 | 7 | 0 |
| **Sex** | Male | 424 | 48 | 46 | 2 |
|  | Female | 135 | 19 | 19 | 0 |
| **Smoking** | Yes | 272 | 40 | 40 | 0 |
|  | No | 287 | 27 | 27 | 0 |
| **HIV status** | HIV positive | 433 | 15 | 14 | 1 |
|  | HIV negative | 126 | 52 | 51 | 1 |
| **Residence** | Urban | 455 | 52 | 52 | 0 |
|  | Rural | 104 | 15 | 15 | 0 |
| **Pet ownership** | Yes | 239 | 25 | 25 | 0 |
|  | No | 320 | 42 | 42 | 0 |

PCR positivity refers to subjects with detectable *Leishmania infantum* DNA by Real-Time PCR. IFAT positivity refers to subjects with IFAT titers ≥1:160 and negative PCR results.

Data derive from clinically tested individuals enrolled through hospital-based surveillance and therefore do not represent population-level prevalence estimates.
