# Supplementary material for: Integrated One Health surveillance of human, canine and sand fly Leishmania infantum transmission in Sicily, southern Italy
Source: Parasit Vectors. 2026 Jul 11;19:431. doi: 10.1186/s13071-026-07520-z (PMC13640383; doi:10.1186/s13071-026-07520-z)
Supplement: Supplementary file 2 [file 13071_2026_7520_MOESM2_ESM.docx]

**Supplementary Table S2.** Canine IFAT results by municipality and province in Sicily between 2022 and 2024.

| Province | Municipality | Dogs tested | IFAT Suspect  (1:40–1:80) |  | IFAT positive (≥1:160) | Seroprevalence (%) | PCR  positive/total |
| --- | --- | --- | --- | --- | --- | --- | --- |
| Palermo | Palermo | 1073 | 123 |  | 139 | 12.95 | 19/104 |
| Palermo | Campofiorito | 24 | 3 |  | 5 | 20.83 | 4/24 |
| Palermo | Caccamo | 23 | 1 |  | 5 | 21.73 | 4/23 |
| Palermo | Ciminna | 14 | 1 |  | 3 | 21.42 | 2/13 |
| Palermo | Misilmeri | 48 | 4 |  | 15 | 31.25 | 10/26 |
| Palermo | Bagheria | 268 | 35 |  | 51 | 19.02 |  |
| Palermo | Trabia | 11 | 4 |  | 4 | 36.36 |  |
| Palermo | Castelbuono | 15 | 2 |  | 3 | 20 | 1/12 |
| Palermo | Petralia Sottana | 180 | 21 |  | 22 | 12.22 | 1/20 |
| Palermo | Termini Imerese | 54 | 10 |  | 11 | 20.37 |  |
| Caltanissetta | Niscemi | 8 | 2 |  | 1 | 12.5 | - |
| Agrigento | Sciacca | 304 | 53 |  | 104 | 34.21 | 26/106 |
| Agrigento | Bivona | 3 | 0 |  | 3 | 100 |  |
| Agrigento | Casteltermini | 13 | 1 |  | 10 | 76.92 | - |
| Agrigento | Cammarata | 220 | 76 |  | 42 | 19.09 | 6/10 |
| Agrigento | Palma di Montechiaro | 20 | 3 |  | 5 | 25 | 0/7 |
| Agrigento | Porto Empedocle | 20 | 5 |  | 5 | 25 | 1/4 |
| Agrigento | Realmonte | 2 | 2 |  | 0 | 0 |  |
| Agrigento | Ravanusa | 153 | 26 |  | 5 | 3.26 |  |
| Agrigento | Agrigento | 332 | 27 |  | 35 | 10.54 | 13/37 |
| Agrigento | Menfi | 42 | 11 |  | 5 | 11.90 | 0/2 |
| Agrigento | Sambuca di Sicilia | 142 | 29 |  | 30 | 21.12 |  |
| Agrigento | Cattolica Eraclea | 77 | 12 |  | 4 | 5.19 |  |
| TOT |  | 3046 | 451 |  | 507 |  |  |

**Legend:** IFAT, indirect immunofluorescence antibody test. IFAT positivity refers to subjects with IFAT titers ≥1:160 Seroprevalence estimates from municipalities with very small sample sizes should be interpreted cautiously.
