# Supplementary material for: Integrated One Health surveillance of human, canine and sand fly Leishmania infantum transmission in Sicily, southern Italy
Source: Parasit Vectors. 2026 Jul 11;19:431. doi: 10.1186/s13071-026-07520-z (PMC13640383; doi:10.1186/s13071-026-07520-z)
Supplement: Supplementary file 3 [file 13071_2026_7520_MOESM3_ESM.docx]

**Table S3.** Standardized sand fly activity and linked decadal meteorological variables during the main entomological season, Sicily, 2022–2024.

| **Year** | **Province** | **Capture period** | **Sampling months** | **Trap-nights, n** | **Positive trap-nights, n (%)** | **Sand flies, n** | **Captures/trap-night** | **Peak capture month/window** | **SIAS station(s) used** | **Mean temperature of matched decades, °C** | **Mean precipitation of matched decades, mm** | **Cumulative precipitation of unique matched decades, mm** | **Mean precipitation in previous 3 decades, mm** |
| --- | --- | --- | --- | --- | --- | --- | --- | --- | --- | --- | --- | --- | --- |
| 2022 | Agrigento | 01 Jun–26 Oct | Jun, Aug, Sep, Oct | 5 | 5 (100.0) | 2,938 | 587.6 | September | Sciacca | 23.9 | 23.4 | 117.0 | 57.6 |
| 2022 | Caltanissetta | 06 May–20 Oct | May–Oct | 17 | 16 (94.1) | 12,308 | 724.0 | September | Caltanissetta | 23.3 | 13.1 | 150.4 | 23.5 |
| 2023 | Agrigento | 18 Jul–30 Oct | Jul–Oct | 15 | 13 (86.7) | 4,124 | 274.9 | September | Sciacca | 25.7 | 2.4 | 26.4 | 6.0 |
| 2023 | Caltanissetta | 05 May–26 Oct | May–Oct | 20 | 19 (95.0) | 13,053 | 652.7 | September–October | Caltanissetta | 22.1 | 12.1 | 135.6 | 32.1 |
| 2023 | Palermo | 04 May–26 Oct | May–Oct | 26 | 2 (7.7) | 5 | 0.2 | July–August, very low activity | Partinico; Termini Imerese | 23.1 | 15.9 | 250.2 | 43.6 |
| 2024 | Agrigento | 17 Jun–27 Aug | Jun–Aug | 13 | 13 (100.0) | 7,981 | 613.9 | July–August | Agrigento Mandrascava; Sciacca | 27.9 | 9.0 | 109.4 | 2.7 |
| 2024 | Caltanissetta | 03 May–11 Oct | May–Oct | 27 | 19 (70.4) | 34,313 | 1,270.9 | June–August | Caltanissetta; Gela | 22.8 | 14.1 | 281.0 | 32.7 |
| 2024 | Palermo | 05 May–17 Oct | May–Oct | 89 | 13 (14.6) | 61 | 0.7 | August–October, very low activity | Alia; Castelbuono; Corleone; Palermo; Partinico; Prizzi; Termini Imerese | 23.8 | 11.2 | 712.2 | 26.7 |

The table includes only trap-nights documented during the main entomological season, defined as May–October. Capture period refers to the first and last documented trapping date included in each province-year stratum. Capture data were standardized as sand flies per documented trap-night. Meteorological variables were linked to each capture date using the corresponding decadal SIAS record from the nearest available station. “Matched decades” refers to the climatic decades containing the capture dates. “Previous 3 decades” refers approximately to the 30 days preceding each capture date. Climate–vector relationships were interpreted as descriptive temporal concordance and not as evidence of direct causal effects. Accordingly, the trap-night totals reported in this table differ from the overall trapping effort values described in the manuscript, which include all trapping activities performed throughout the entire study period.
